# Evaluation of the Clinical Characteristics and Survival of Pediatric Patients with Invasive Aspergillosis: A 12-year Retrospective Cohort Study

**DOI:** 10.1007/s11046-026-01078-1

**Published:** 2026-05-13

**Authors:** Azer Karaman, Ali Bulent Cengiz, Kubra Aykac, Osman Oguz Demir, Dolunay Gülmez, Sevtap Arikan-Akdagli, Yasemin Ozsurekci

**Affiliations:** 1 Department of Pediatrics, Faculty of Medicine, Hacettepe University, Ankara, Türkiye; 2 Department of Pediatric Infectious Diseases, Faculty of Medicine, Hacettepe University, Ankara, Türkiye; 3 Department of Medical Microbiology, Faculty of Medicine, Hacettepe University, Ankara, Türkiye

**Keywords:** Children, Invasive aspergillosis, Aspergillus species, Treatment outcome

## Abstract

**Background:**

Invasive aspergillosis (IA) is a major cause of morbidity and mortality in immunocompromised children. Pediatric data are limited, and treatment guidelines often rely on adult studies. This study aimed to assess clinical characteristics and treatment outcomes of pediatric IA.

**Methods:**

We conducted a retrospective cohort study of pediatric patients (0–18 years) diagnosed with IA at a tertiary center in Türkiye between 2010 and 2022. Data on demographics, underlying conditions, *Aspergillus* species, antifungal susceptibility, treatment regimens, and outcomes were analyzed. Survival analysis was performed using Kaplan–Meier curves.

**Results:**

Fifty-five children met criteria for proven (69%) or probable (31%) IA after excluding 22 possible cases. Median age was 8.6 years; mortality did not differ significantly by age, sex, or underlying condition. Hematologic malignancies were the most common comorbidity (35%). *Aspergillus fumigatus* was the predominant species (57%), and no antifungal resistance was detected. Classical risk factors; central venous catheter use (53%), neutropenia (51%), and recent chemotherapy (35%) were frequent but showed no association with mortality. Lung involvement was the most common presentation (71%), followed by sinus (31%) and central nervous system involvement (13%). Thirty-day mortality was 11%, 12-week mortality was 20% (11/55), and overall mortality reached 29%.

**Conclusions:**

Pediatric IA remains associated with substantial morbidity and mortality, although outcomes in this cohort were more favorable than those reported in many prior pediatric series. No single clinical, microbiological, or radiological factor reliably predicted mortality, underscoring the complexity of risk stratification. Early diagnosis and timely antifungal therapy remain essential, and multicenter prospective studies are needed to optimize treatment approaches.

## Introduction

Invasive aspergillosis (IA) remains a challenging problem in pediatric healthcare, particularly among immunocompromised children [1]. Globally, life-threatening fungal infections affect more than two million people each year, highlighting the substantial burden imposed by invasive mold diseases [2]. The disease most commonly affects patients with hematological malignancies, those undergoing hematopoietic stem cell or solid organ transplantation, or children receiving intensive immunosuppressive therapies [3, 4]. The most common causative species of invasive aspergillosis in children include *Aspergillus fumigatus*, followed by *Aspergillus flavus, Aspergillus terreus*, and less frequently *Aspergillus niger* [3]. In recent years, medical advances that prolong the survival of immunocompromised patients have paradoxically increased their susceptibility to invasive fungal infections, including IA [2, 5]. While IA has been extensively studied in adult populations, pediatric data are relatively scarce. Consequently, pediatric treatment guidelines often extrapolate from adult studies, despite the distinct physiological and pharmacokinetic characteristics of children. Mortality rates for pediatric IA remain high, often exceeding 25%, underscoring the urgent need for improved diagnostic and therapeutic approaches [6–8]. Key factors that influence IA outcomes include the degree of immunosuppression, the timeliness of diagnosis, and the adequacy of antifungal therapy. The critical question remains which management strategies are most effective in reducing mortality rates among pediatric patients.

While monotherapy is often preferred in the first-line treatment of IA in pediatric populations, voriconazole and liposomal amphotericin B are the most commonly used agents for monotherapy, while combination regimens frequently include an azole or amphotericin B combined with an echinocandin such as caspofungin. Combination therapy is often considered in cases of failure of conventional first-line monotherapy [4, 9–12]. Recent studies have demonstrated the efficacy of combining echinocandins with azoles or amphotericin B in improving clinical outcomes and reducing mortality [12, 13]. Despite this, evidence supporting combination therapy in pediatric populations remains limited, and data on its comparative efficacy versus monotherapy are inconsistent. Furthermore, existing literature rarely addresses outcomes based on underlying conditions beyond hematologic malignancies, such as solid tumors or primary immunodeficiencies.

In this study, we aimed to fill these knowledge gaps by retrospectively evaluating the clinical characteristics, risk factors, pathogen distribution, and treatment outcomes of pediatric patients with IA treated at a tertiary referral center over a 12-year period. We specifically compared clinical responses and mortality outcomes among those who received monotherapy, combination therapy, or salvage regimens. Our findings are intended to inform future prospective studies and help refine clinical decision-making in pediatric IA management.

## Method

### Study Design and Setting

This was a retrospective cohort study conducted at the Department of Pediatric Infectious Diseases, Hacettepe University Faculty of Medicine, a tertiary referral center in Ankara, Türkiye. The study period spanned from January 2010 to December 2022.

### Patient Selection and Data Collection

We included all pediatric patients (aged 0–18 years) diagnosed with invasive aspergillosis (IA) during the study period. IA was classified according to the revised definitions of the European Organization for Research and Treatment of Cancer and the Mycoses Study Group Education and Research Consortium (EORTC/MSGERC) into possible, probable, and proven cases [14]. Data were extracted from hospital records and included demographics (age, gender), underlying diseases (hematologic malignancy, oncologic malignancy, primary immunodeficiency, etc.) diagnostic classification of IA, *Aspergillus* species isolated, radiological and microbiological findings, antifungal treatment regimen, clinical outcomes, and mortality at 30 days and 12 weeks. The study was approved by the ethical committee of Hacettepe University (GO 23/245). This study was conducted without the requirement for informed consent. This determination was based on the use of existing medical records without additional patient interventions, and all data were deidentified to ensure patient privacy.

### Microbiological Evaluation

*Aspergillus* species isolated from cultures of clinical specimens (bronchoalveolar lavage (BAL) fluid, and tissue samples) were identified to species level via matrix assisted laser desorption time of flight mass spectrophotometry (MALDI-TOF MS, Bruker Daltronics, Germany). Antifungal susceptibility testing of the isolates was performed in compliance with the European Committee on Antimicrobial Susceptibility Testing (EUCAST) guidelines and the results were evaluated according to available breakpoints for each species antifungal combination. Voriconazole, itraconazole and amphotericin B susceptibilities were tested using EUCAST reference microdilution method (E.DEF 9.4) and echinocandin (micafungin and anidulafungin) susceptibility was tested by agar screening [14, 15].

### Diagnostic evaluation

A galactomannan positive result is defined as an optical density index (ODI) of ≥ 0.5 in serum, ≥ 1 in BAL, and an optical density index of ≥ 0.5 in CSF (7). The chest computed tomography reports of patients were reviewed at the time of IA diagnosis according to the guideline [16].

### Treatment Categorization

Antifungal treatment regimens were classified into three categories: a) Monotherapy: Use of a single antifungal agent; b) Combination therapy: Concurrent use of two or more antifungal agents; c) Salvage therapy: Initiation of antifungal treatment after failure of prior regimen or intolerant of the initial treatment [4].

### Outcome Definition

Treatment response was categorized as complete response, failure, or death. Complete responses were characterized by the resolution of all clinical signs and symptoms, as well as a reduction of over 90% in the lesions related to invasive aspergillosis that were observable through radiological imaging. Partial responses, on the other hand, were indicated by clinical improvement and more than a 50% improvement in radiological findings [17]. Mortality was assessed at two timepoints: 30-day and 12-week follow-up from IA diagnosis [10]. Overall survival analysis was also conducted.

### Statistical analysis

All statistical analyses were performed using RStudio. Continuous variables, such as age and duration of antifungal therapy, were summarized using medians and interquartile ranges (IQRs) due to their non-normal distribution. Categorical variables, including underlying conditions, microbiological findings, organ involvement, and treatment regimens, were presented as counts and percentages. Group comparisons between survivors and non-survivors were conducted using the Mann–Whitney U test for continuous variables and Fisher’s exact or Pearson’s χ^2^ tests for categorical variables, depending on expected cell counts. Survival probabilities up to 12 weeks were estimated using Kaplan–Meier curves. Differences in survival according to diagnostic category (proven vs. probable IA) and underlying disease groups were assessed using the log-rank test. All statistical tests were two-sided, and a *p*-value < 0.05 was considered statistically significant.

## Results

### Patient Characteristics and Mortality

After excluding 22 cases classified as possible invasive fungal disease, 55 children with proven or probable invasive aspergillosis were included. By week 12, 11 patients (20%) had died, while 44 (80%) survived (Table 1).

**Table 1:** Demographic and clinical data of patients according to mortality on week-12

	Total N = 55^1^	Alive N = 44^1^	Exitus N = 11^1^	*p*-value^2^
Age	8.6 (2.9—14.3)	6.8 (2.1—13.9)	11.0 (8.6—15.5)	0.123
Gender (Male)	23 (42)	18 (41)	5 (45)	> 0.999
*Underlying diseases*				0.801
Hematologic malignancy	19 (35)	15 (34)	4 (36)	
Oncologic malignancy	6 (11)	4 (9)	2 (18)	
Aplastic anemia	4 (7)	3 (7)	1 (9)	
PID	13 (24)	12 (27)	1 (9)	
HSCT	7 (13)	5 (11)	2 (18)	
Other	6 (11)	5 (11)	1 (9)	
*Diagnosis*				0.422
Proven	38 (69)	32 (73)	6 (55)	
Probable	17 (31)	12 (27)	5 (45)	
*Aspergillus species*				0.451
*A. flavus/oryzae*	10/ 30 (33)	7/ 26 (27)	3/ 4 (75)	
*A. fumigatus*	17/ 30 (57)	16/ 26 (62)	1/ 4 (25)	
*A. fumigatus* and *A. niger*	1/ 30 (3)	1/ 26 (4)	0/ 4 (0)	
*A. niger*	1/ 30 (3)	1/ 26 (4)	0/ 4 (0)	
*A. terreus*	1/ 30 (3)	1/ 26 (4)	0/ 4 (0)	
*Risk factors at last 4 weeks*				
Central venous catheter	29 (53)	21 (48)	8 (73)	0.251
Neutropenia	28 (51)	21 (48)	7 (64)	0.544
Total parenteral nutrition	6 (11)	4 (9)	2 (18)	0.746
IMV	3 (5)	2 (5)	1 (9)	> 0.999
Surgery	2 (4)	2 (5)	0	> 0.999
Hemodialysis	4 (7)	3 (7)	1 (9)	> 0.999
PICU admission	8 (15)	6 (14)	2 (18)	> 0.999
Bacterial infection	13 (24)	10 (23)	3 (27)	> 0.999
Recent chemotherapy	19 (35)	14 (32)	5 (45)	0.620
*Duration of treatment*	58 (34—109)	79 (44—113)	30 (13—56)	< 0.001
*Treatment group*				0.220
Monotherapy	20 (36)	16 (36)	4 (36)	
Combined	26 (47)	19 (43)	7 (64)	
Sequential	9 (16)	9 (20)	0	
*Outcome*				< 0.001
Complete response	39 (71)	39 (89)	0	
Partial response	0	0	0	
Stable	0	0	0	
Failure of therapy	16 (29)	5 (11)	11 (100)	
Indeterminate	0	0	0	
*Mortality at day30*	6 (11)	0	6 (55)	< 0.001
*Overall mortality*	16 (29)	5 (11)	11 (100)	< 0.001

^1^Median (Q1—Q3); n / N (%), ^2^Wilcoxon rank sum test; Pearson’s Chi-squared test; Fisher’s exact test, HSCT: Hematopoietic stem cell transplantation, PID: Primary immunodeficiency, Other:Solid organ transplant, Seconder immunodeficiency. CNS: Central nervous system, PICU: Pediatric Intensive Care Unit, IMV: Invasive mechanical ventilation

The median age of the cohort was 8.6 years (IQR 2.9–14.3). Survivors had a median age of 6.8 years, whereas non-survivors had a median age of 11.0 years, although this difference was not statistically significant (*p* = 0.123). Males accounted for 42% (23/55) of the cohort, with similar proportions among survivors (41%) and non-survivors (45%) (*p* > 0.999).

Hematologic malignancy was the most common underlying condition (35%, 19/55), with nearly identical distribution between survivors (34%) and non-survivors (36%) (*p* = 0.801). Oncologic malignancy was present in 11% (6/55) of patients. Although numerically higher among non-survivors (18% vs. 9%), this difference did not reach statistical significance (*p* = 0.801). Primary immunodeficiency was observed in 24% of survivors and 9% of non-survivors.

Proven IA accounted for 69% (38/55) of cases and probable IA for 31% (17/55), without a significant association with mortality (*p* = 0.422).

### Microbiological Findings

Species identification was available for 30 patients. *A. fumigatus* was the most frequent (57%, 17/30), followed by *A. flavus/oryzae* (33%, 10/30). Rare isolates included mixed *A. fumigatus* + *A. niger*, *A. niger*, and *A. terreus* (each 3%, 1/30). Species distribution did not differ significantly between survivors and non-survivors (*p* = 0.451). In vitro resistance was not detected in any of the *Aspergillus* isolates against the antifungal drugs tested (Table 2).

**Table 2:** Azole and amphotericin B minimum inhibitory concentration (MIC) values (mg/l), echinocandin agar screening test results* and available susceptibility cathegories of *Aspergillus* isolates using EUCAST methodology

Patient No	*Aspergillus* species	Voriconazole	Itraconazole	Amphotericin B	Micafungin	Anidulafungin
1	*Aspergillus fumigatus*	0.5 (S)	0.5 (S)	1 (S)	S	S
2	*Aspergillus fumigatus*	0.5 (S)	0.5 (S)	0.5 (S)	S	S
3	*Aspergillus fumigatus*	0.5 (S)	0.5 (S)	0.5 (S)	S	S
4	*Aspergillus fumigatus*	0.5 (S)	0.25 (S)	1 (S)	S	S
5	*Aspergillus terreus*	0.5	0.5 (S)	1	S	S
6	*Aspergillus flavus/oryzae*	0.5	0.25 (S)	0.5 (S)	S	S
7	*Aspergillus fumigatus*	0.5 (S)	0.5 (S)	1 (S)	S	S
8	*Aspergillus fumigatus*	0.5 (S)	0.5 (S)	0.5 (S)	S	S
9	*Aspergillus fumigatus*	0.5 (S)	0.5 (S)	1 (S)	S	S
10	*Aspergillus fumigatus*	0.5 (S)	0.25 (S)	1 (S)	S	S
11	*Aspergillus fumigatus*	0.5 (S)	0.5 (S)	1 (S)	S	S
	*Aspergillus niger*	1	1	1 (S)	S	S
12	*Aspergillus flavus/oryzae*	1	0.25 (S)	1	S	S
13	*Aspergillus flavus/oryzae*	1	0.25 (S)	2	S	S
14	*Aspergillus flavus/oryzae*	0.5	0.25 (S)	1	S	S
15	*Aspergillus fumigatus*	0.5 (S)	0.25 (S)	0.5 (S)	S	S
16	*Aspergillus fumigatus*	0.5 (S)	0.5 (S)	1 (S)	S	S
17	*Aspergillus fumigatus*	0.5 (S)	0.5 (S)	1 (S)	S	S
18	*Aspergillus flavus/oryzae*	0.5	0.5 (S)	1	S	S
19	*Aspergillus flavus/oryzae*	0.5	0.5 (S)	1	S	S
20	*Aspergillus fumigatus*	1 (S)	0.5 (S)	0.5 (S)	S	S
21	*Aspergillus fumigatus*	0.5 (S)	0.25 (S)	0.5 (S)	S	S
22	*Aspergillus flavus/oryzae*	1	0.5 (S)	0.5	S	S
23	*Aspergillus flavus/oryzae*	0.5	0.5 (S)	1	S	S
24	*Aspergillus fumigatus*	1 (S)	1 (S)	1 (S)	S	S
25	*Aspergillus fumigatus*	0.5 (S)	0.5 (S)	1 (S)	S	S
26	*Aspergillus fumigatus*	0.5 (S)	0.5 (S)	0.5 (S)	S	S
27	*Aspergillus flavus/oryzae*	1	0.25 (S)	1	S	S
28	*Aspergillus flavus/oryzae*	0.5	0.25 (S)	1	S	S
29	*Aspergillus fumigatus*	0.5 (S)	0.5 (S)	1 (S)	S	S
30	*Aspergillus flavus/oryzae*	0.5	0.5 (S)	1	S	S

S, Susceptible;

*Compact, non-fluffy colonies were observed after 48 h of incubation for all isolates belonging to different *Aspergillus* species using the agar screening method developed for *Aspergillus* and as exemplified by *A. fumigatus*

### Risk Factors, Clinical and Radiological Features

Recent risk factors were common in the cohort, with central venous catheter (CVC) use in 53% (29/55), neutropenia in 51% (28/55), and recent chemotherapy in 35% (19/55) of patients; however, none of these variables showed a significant association with mortality. Other factors including total parenteral nutrition (11%), invasive mechanical ventilation (5%), hemodialysis (7%), PICU admission (15%), and bacterial infection (24%) were less frequent and occurred at similar rates among survivors and non-survivors. The median duration of antifungal therapy in the entire cohort was 58 days (IQR 34–109).

**Fig. 1:**
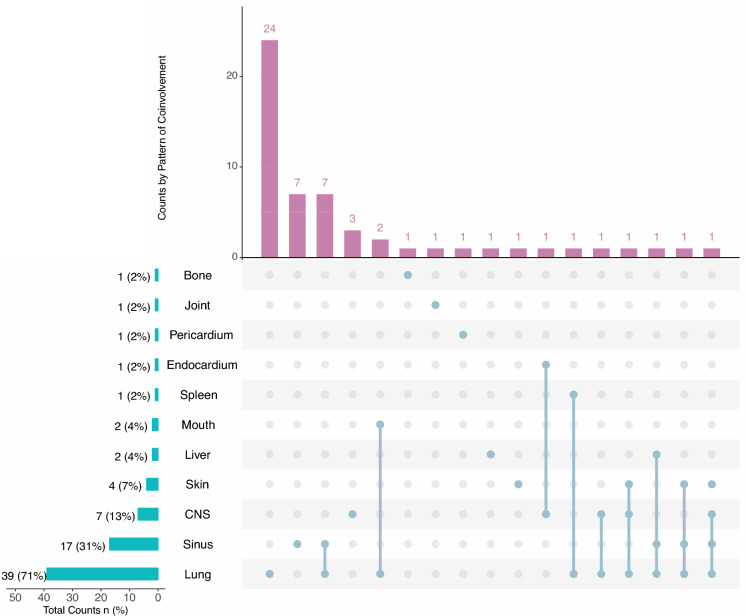
Anatomical distribution of organ involvement among 55 children with proven or probable invasive aspergillosis. Pulmonary involvement was the most frequent presentation (71%), followed by sinus (31%) and central nervous system involvement (13%)

Patterns of organ involvement among the 55 children with invasive aspergillosis are shown in Fig. 1. Lung involvement was the most frequent finding (39 patients, 71%), followed by sinus involvement (17 patients, 31%) and central nervous system involvement (7 patients, 13%). Skin (7%), liver (4%), mouth (4%), spleen (2%), endocardium (2%), pericardium (2%), joint (2%), and bone (2%) were less commonly affected. Co-involvement patterns varied, with the most frequent combination involving the lungs (n = 24), followed by lung–sinus (n = 7) and lung–CNS (n = 7) involvement.

**Fig. 2:**
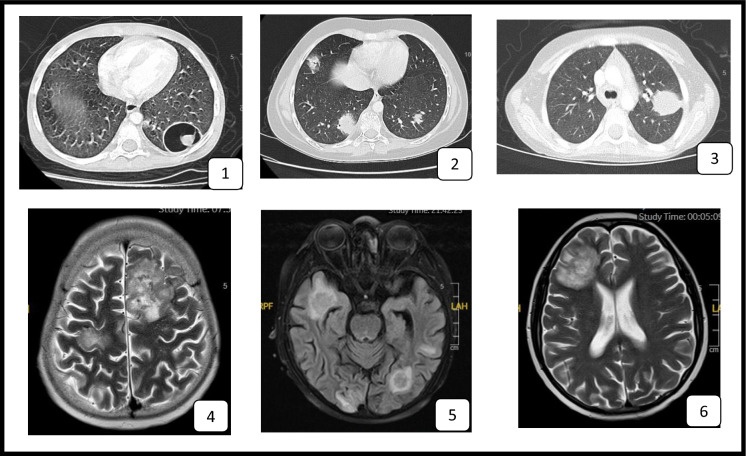
Representative radiological findings of invasive aspergillosis in six pediatric patients, demonstrating pulmonary and central nervous system involvement Patients 1–3 (Pulmonary IA): Patient 1: Chest CT showing a thick-walled cavitary lesion in the right lower lobe. Patient 2: Chest CT demonstrating multiple bilateral nodular opacities. Patient 3: Chest CT revealing a solitary, well-defined pulmonary nodule in the right upper lobe surrounded by a subtle halo of ground-glass opacity, consistent with early angioinvasive disease. Patients 4–6 (CNS IA): Patient 4: Axial T2-weighted brain MRI showing a heterogeneous right frontal cortical lesion with surrounding edema and areas of hemorrhage, compatible with fungal vasculitis or infarction. Patient 5: Axial FLAIR MRI demonstrating bilateral temporal cortical lesions with surrounding edema and mass effect, suggestive of disseminated cerebral aspergillosis. Patient 6: Axial T2-weighted MRI illustrating a large heterogeneous lesion with perifocal edema in the left frontal lobe, consistent with a fungal abscess.

Representative radiological findings from six children with pulmonary or central nervous system involvement are shown in Fig. 2, illustrating the spectrum of imaging abnormalities observed in invasive aspergillosis.

### Treatment Regimens and Outcomes

All patients received systemic antifungal therapy, including monotherapy (36%, 20/55), combination therapy (47%, 26/55), and sequential therapy (16%, 9/55). The most commonly used antifungal agents for monotherapy were voriconazole (n = 4) and liposomal amphotericin B (n = 16). Combination therapy most frequently included liposomal amphotericin B combined with voriconazole (n = 14), as well as regimens combining azoles with echinocandins such as caspofungin or micafungin. Sequential therapy generally involved switching between azoles and amphotericin B formulations (n = 11).

The distribution of treatment regimens did not differ significantly between survivors and non-survivors (monotherapy: 36% vs. 36%; combination therapy: 43% vs. 64%; sequential therapy: 20% vs. 0%; p = 0.220). A complete clinical response was observed in 71% (39/55) of patients, while 29% (16/55) experienced therapy failure. All non-survivors met criteria for treatment failure. Thirty-day mortality was 11% (6/55), twelve-week mortality was 20% (11/55) and overall mortality was 29% (16/55).

**Fig. 3:**
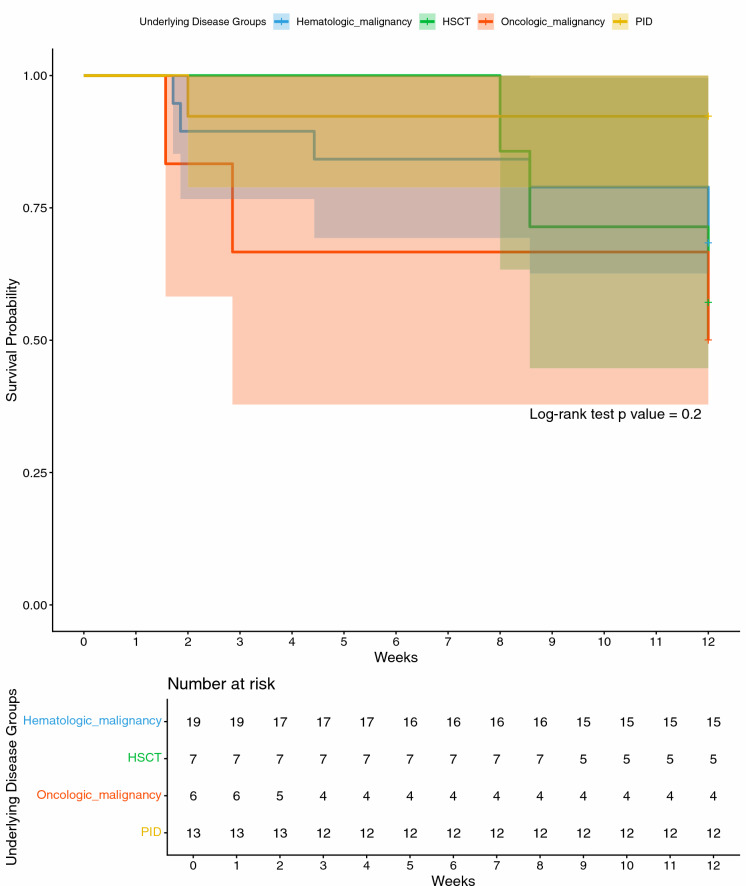
Kaplan–Meier analysis illustrating 12-week overall survival among children with invasive aspergillosis, stratified by underlying disease category: hematologic malignancy, oncologic malignancy, hematopoietic stem cell transplantation (HSCT), and primary immunodeficiency (PID). Survival probabilities did not differ significantly across groups (log-rank p = 0.20). The shaded areas represent 95% confidence intervals. Number-at-risk tables for each disease group are displayed below the plot

Kaplan–Meier survival analysis comparing underlying disease groups, including hematologic malignancy, oncologic malignancy, hematopoietic stem cell transplantation (HSCT), and primary immunodeficiency (PID) showed no significant difference in overall survival (log-rank *p* = 0.20) (Fig. 3).

## Discussion

In this 12-year single-center cohort, we evaluated the clinical characteristics, microbiological patterns, and outcomes of invasive aspergillosis in children, using updated diagnostic classifications restricted to proven and probable disease [14]. The overall 12-week mortality rate was 20%, lower than previously reported ranges in pediatric IA (6, 7, 18). Hematologic malignancies remained the most common underlying condition, although IA also occurred in children with oncologic malignancies and primary immunodeficiencies. Survival did not differ significantly between proven and probable IA, and treatment regimens showed similar distributions among survivors and non-survivors. Together, these findings reflect the complex and multifactorial nature of IA in pediatric patients and highlight ongoing challenges in early recognition and management.

In recent years, there has been a significant increase in pediatric patients at risk due to the widespread use of immunosuppressive drugs and the prolonged survival of patients with immune deficiencies [1, 3]. Various factors contribute to the emergence of IA, including organ transplants, chronic conditions such as chronic lung diseases, immunosuppressive therapies, frequent hospitalizations, and the increased number of invasive procedures, as well as the human immunodeficiency virus epidemic. Moreover, IA can develop in immunocompetent individuals through the influence of factors that predispose them to these infections.

Studies conducted in different regions have shown that the incidence of IA ranges between 14.1 and 27.2 cases per 100,000, and they are associated with both high morbidity and mortality rates. It is reported that more than 2 million people worldwide are affected by life-threatening fungal infections annually, contributing to a significant global disease burden [2]. Reported mortality rates in pediatric IA vary widely across studies. In our cohort, the 12-week mortality was 20% and overall mortality reached 29%, both of which remain at or below the ranges described in many previous pediatric series. [6–8, 18]. Identifying prognostic factors could aid in determining which patients need risk modification and/or more intensive treatment. Several studies highlight the impact of underlying immunosuppression, comorbid conditions, and disseminated infection on survival [5, 19]. In a study conducted in our country, it was reported that 27% of relapsed or refractory leukemias treated with cytarabine resulted in death [20]. Similarly, a study by Harris et al. reported that 23% of refractory leukemia cases died due to invasive fungal infections [21]. In the current study, all-cause mortality was 27.2% within 12 weeks of the initial diagnosis and the majority of deaths (66.6%) occurred within 30 days of the IA diagnosis, consistently with previous reports [18, 22, 23].

In alignment with existing literature, our study identifies patients who have undergone hematopoietic stem cell transplantation (HSCT) as one of the most critical cohorts at risk for developing IA. Most studies to date have identified neutropenia as a significant factor influencing mortality, highlighting the crucial role of immune system recovery in HSCT patients with IA [5, 24]. Several studies, including the work of Baddley et al. [25], have reported that proven IA is more frequently observed among non-survivors, often interpreted as a marker of more advanced disease or delays in diagnosis. Likewise, previous experimental and observational studies have suggested that proven IA, compared with probable IA, may be associated with poorer outcomes [5, 19]. This observation is intuitive, as probable IA cases generally involve less diagnostic certainty. In contrast, we observed that a proven diagnosis of IA was associated with a better prognosis. This likely reflects both the benefits of early and accurate diagnosis and the possibility that patients with proven IA had more definitive management based on microbiological confirmation. In contrast, patients classified under possible IA had significantly worse outcomes, likely due to diagnostic uncertainty and delays in appropriate therapy.

The type of causative agent may also influence mortality rates [26]. Although *A. fumigatus* was the predominant species overall, *A. flavus/oryzae* was more frequently isolated in non-survivors. While the difference was not statistically significant, this observation has a possibility suggesting that *A. flavus/oryzae* infections may be more aggressive in certain clinical contexts. Further studies with larger cohorts are likely needed to elucidate this issue more clearly. Such studies may help tailor treatment strategies based on causative species and support timely intervention, especially regarding the role of combination therapy.

There has been substantial improvement in pediatric IA outcomes with the introduction of liposomal amphotericin B and newer antifungal classes such as triazoles and echinocandins, combined with increased clinical awareness 27–29]. Nonetheless, mortality remains high, and uncertainty persists regarding the relative benefits of monotherapy versus combination regimens, largely due to the retrospective nature of available studies. Combination therapy is frequently considered in children with hematologic malignancies or progressive disease, although evidence supporting its superiority remains inconsistent [30–32]. Conversely, other pediatric reports noted that all fatal cases had received monotherapy [19]. In our cohort, combination therapy was more common among non-survivors but did not reach statistical significance, likely reflecting treatment escalation in more severely ill patients rather than an adverse effect of the regimen itself.

This study has several limitations. First, its retrospective, single-center design may limit the generalizability of the findings, as clinical practices, diagnostic resources, and patient populations can vary across institutions. Second, although we applied updated EORTC/MSG definitions, distinguishing proven from probable IA remains challenging in pediatric patients, particularly in those for whom invasive diagnostic procedures are not feasible. Third, despite spanning 12 years, the sample size, especially for subgroup analyses was relatively small, reducing statistical power to detect differences in risk factors or treatment effects. Additionally, treatment decisions were not protocol-driven but individualized according to clinical judgement, introducing potential confounding, especially in evaluating the impact of antifungal regimens. Finally, the study did not assess radiologic response patterns or therapeutic drug monitoring data, both of which could provide further insight into disease progression and treatment outcomes.

Despite these limitations, the strength of our study lies in its ability to provide valuable insights into the factors associated with mortality in pediatric IA within a large tertiary care center. This contribution, although modest, helps illuminate the clinical landscape of IA in children both in our country and potentially in similar settings worldwide.

## Conclusion

In this 12-year pediatric cohort, invasive aspergillosis remained a serious infection with substantial morbidity and mortality, although outcomes were more favorable than those reported in many earlier series. Most patients had multiple risk factors, yet no single clinical or microbiological variable reliably predicted mortality, underscoring the complexity of risk stratification in children. Treatment responses were generally high, and mortality was not associated with the choice of monotherapy, combination therapy, or sequential therapy. These findings highlight the importance of early recognition, timely initiation of antifungal therapy, and continued vigilance across diverse immunocompromised pediatric populations. Larger, multicenter prospective studies are needed to refine prognostic indicators and optimize management strategies for pediatric invasive aspergillosis.

## Data Availability

The data that support the findings of this study are available on request from the corresponding author. The data are not publicly available due to privacy or ethical restrictions.
